# Supplementary material for: A new pharmacodynamic approach to study antibiotic combinations against enterococci in vivo: Application to ampicillin plus ceftriaxone
Source: PLoS One. 2020 Dec 8;15(12):e0243365. doi: 10.1371/journal.pone.0243365 (PMC7723291; doi:10.1371/journal.pone.0243365)
Supplement: S1 Fig — CRO monotherapy was completely ineffective against E. faecalis in doses up to 200 mg/kg/day. Regression line is shown dotted because Hill’s sigmoid model did not fit to the data. (DOCX) [file pone.0243365.s001.docx]

**S1 Fig . *In vivo* pharmacodynamics of CRO monotherapy vs. *E. faecalis* ATCC 29212**


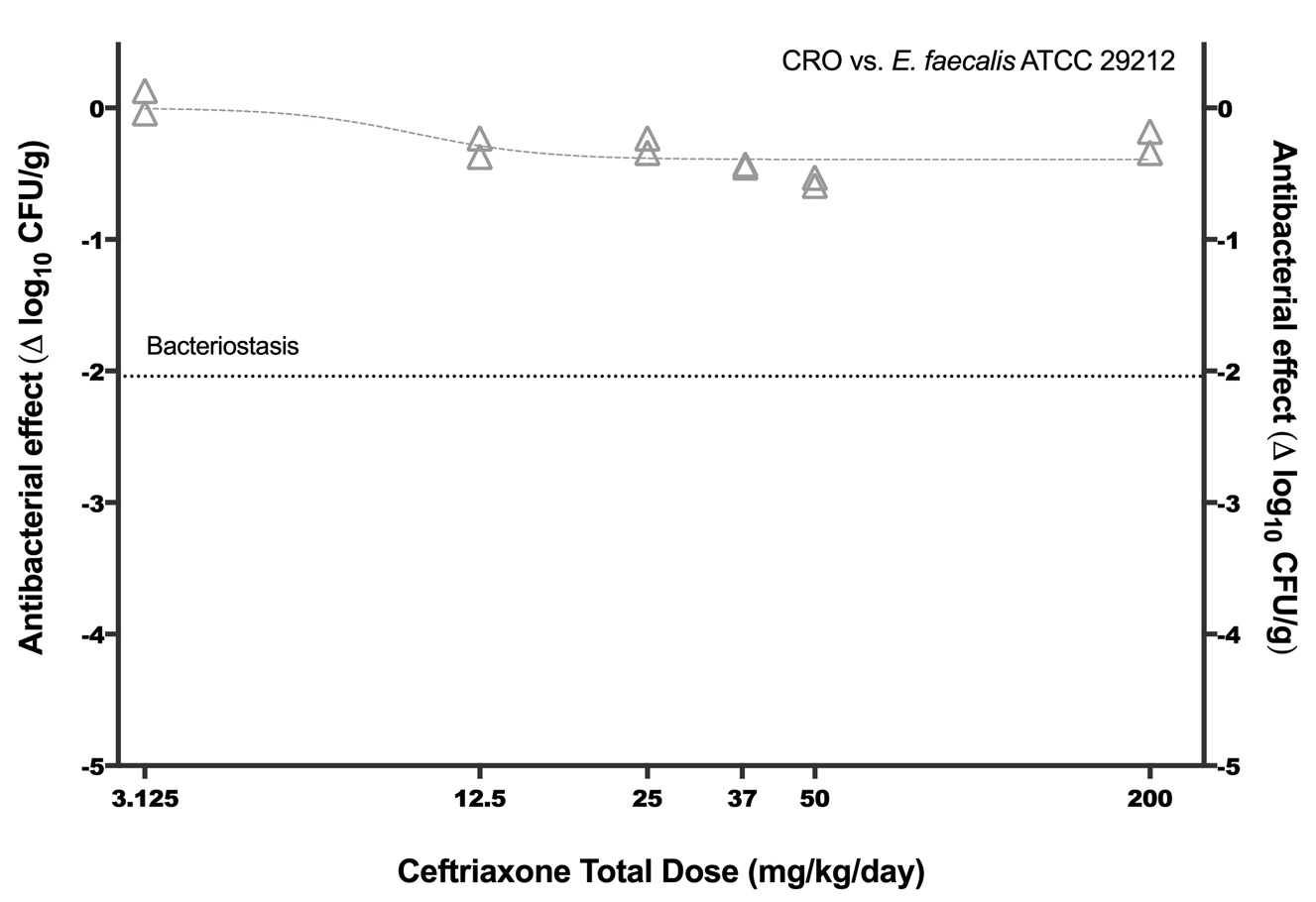


CRO monotherapy was completely ineffective against *E. faecalis* in doses up to 200 mg/kg/day. Regression line is shown dotted because Hill’s sigmoid model did not fit to the data.
