# Supplementary material for: A new pharmacodynamic approach to study antibiotic combinations against enterococci in vivo: Application to ampicillin plus ceftriaxone
Source: PLoS One. 2020 Dec 8;15(12):e0243365. doi: 10.1371/journal.pone.0243365 (PMC7723291; doi:10.1371/journal.pone.0243365)
Supplement: S2 Fig — The parameters BD, 1LKD and 2LKD are shown in Table 3 of the paper. (DOCX) [file pone.0243365.s002.docx]

**S2 Fig. *In vivo* pharmacodynamics of AMP monotherapy vs. *E. faecalis* ATCC 51299**

**
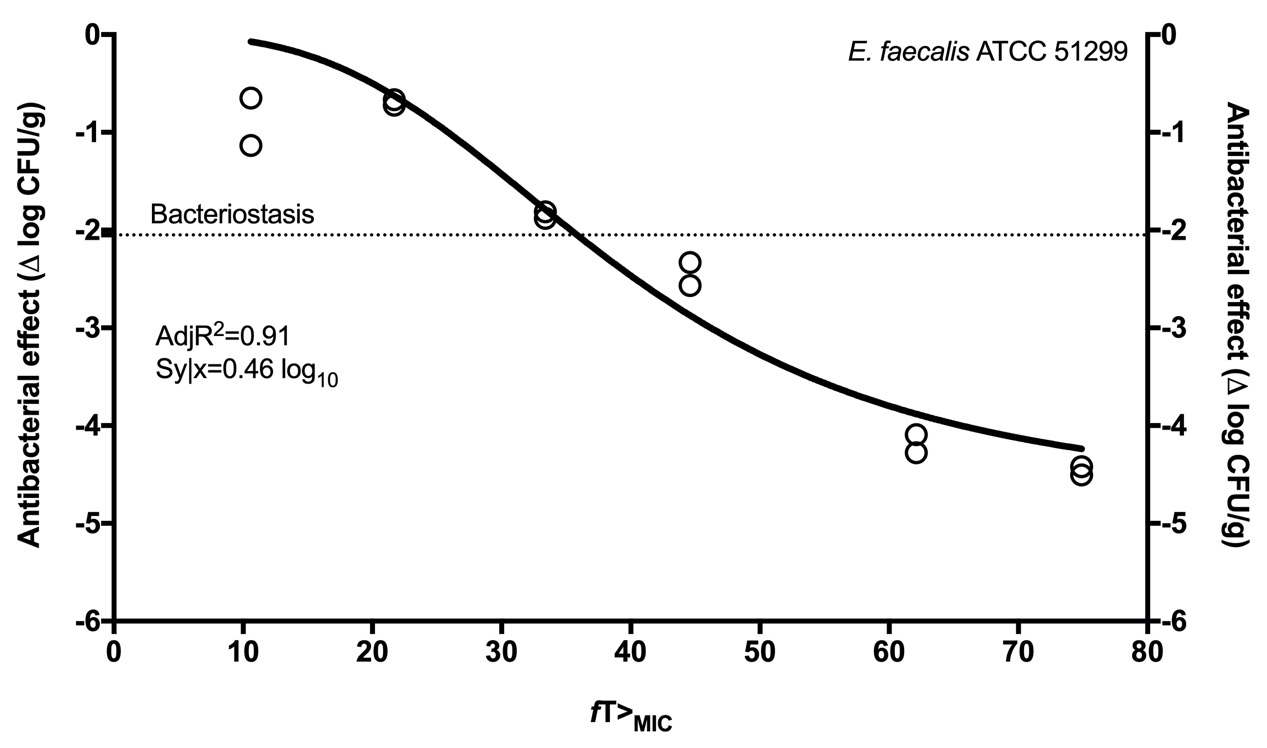
**

The parameters BD, 1LKD and 2LKD are shown in Table 3 of the paper.
