# Supplementary material for: A new pharmacodynamic approach to study antibiotic combinations against enterococci in vivo: Application to ampicillin plus ceftriaxone
Source: PLoS One. 2020 Dec 8;15(12):e0243365. doi: 10.1371/journal.pone.0243365 (PMC7723291; doi:10.1371/journal.pone.0243365)
Supplement: S4 Fig — PK profile in a typical patient weighing 70 kg and with a creatinine clearance of 71 mL/min. The total serum concentration of AMP along 24 hours is displayed (protein binding is 20%). (DOCX) [file pone.0243365.s004.docx]

**S4 Fig. Simulated human PK profile of intravenous AMP 500 mg every 6 hours.**

PK profile in a typical patient weighing 70 kg and with a creatinine clearance of 71 ml/min. The total serum concentration of AMP along 24 hours is displayed (protein binding is 20%).
