# Supplementary material for: A new pharmacodynamic approach to study antibiotic combinations against enterococci in vivo: Application to ampicillin plus ceftriaxone
Source: PLoS One. 2020 Dec 8;15(12):e0243365. doi: 10.1371/journal.pone.0243365 (PMC7723291; doi:10.1371/journal.pone.0243365)
Supplement: S1 Table — (DOCX) [file pone.0243365.s006.docx]

| **S1 Table. *f*T_>MIC_ of AMP doses used against enterococci in mice** | | | | | | | |  |
| --- | --- | --- | --- | --- | --- | --- | --- | --- |
|  |  |  |  |  |  |  |  |  |
|  |  |  |  |  |  |  |  |  |
|  |  | *f*T_>MIC_ (%) * | | | | | |  |
| AMP dose (mg/kg/day) |  | *E. faecalis* ATCC 29212 | | *E. faecalis* ATCC 51299 (VanB) | | *E. faecium* ATCC 19434 | |  |
|  |  |  |  |  |  |  |  |  |
|  |  |  |  |  |  |  |  |  |
| 2400 |  | 69.0 | | 74.9 | | 74.9 | |  |
| 1200 |  | 62.1 | | 69.0 | | 69.0 | |  |
| 600 |  | 54.1 | | 62.1 | | 62.1 | |  |
| 300 |  | 44.6 | | 54.1 | | 54.1 | |  |
| 225 |  | 40.1 | | 50.4 | | 50.4 | |  |
| 150 |  | 33.4 | | 44.6 | | 44.6 | |  |
| 75 |  | 21.7 | | 33.4 | | 33.4 | |  |
| 37.5 |  | 10.5 | | 21.7 | | 21.7 | |  |
| 18.75 |  | 1.7 | | 10.6 | | 10.6 | |  |
| 9.37 |  | 0.0 | | 1.7 | | 1.7 | |  |
| *MIC: ATCC 29212, 1 mg/L; ATCC 51299, 0.5 mg/L and ATCC 19434, 0.5 mg/L. | | | | | | | |  |
|  |  |  |  |  |  |  |  |  |
