# Supplementary material for: A new pharmacodynamic approach to study antibiotic combinations against enterococci in vivo: Application to ampicillin plus ceftriaxone
Source: PLoS One. 2020 Dec 8;15(12):e0243365. doi: 10.1371/journal.pone.0243365 (PMC7723291; doi:10.1371/journal.pone.0243365)
Supplement: S2 Table — (DOCX) [file pone.0243365.s007.docx]

| **S2 Table. % *f*T_>MIC_ of AMP doses used against enterococci in humans.** | | | | |  |
| --- | --- | --- | --- | --- | --- |
|  |  |  |  |  |  |
| **MIC (mg/L)** |  | ***f*T_>MIC_ (%)** | | |  |
|  |  | **AMP dose** | | |  |
|  |  | 500 mg q 6 h |  | 2000 mg q 4 h |  |
| 0.5 |  | 100 |  | 100 |  |
| 1 |  | 88.0 |  | 100 |  |
| 2 |  | 61.8 |  | 100 |  |
| 4 |  | 39.3 |  | 100 |  |
| 8 |  | 23.4 |  | 98.0 |  |
| 16 |  | 11.7 |  | 63.0 |  |
| 32 |  | 0.0 |  | 37.3 |  |
| 64 |  | 0.0 |  | 18.7 |  |
| 128 |  | 0.0 |  | 2.2 |  |
| 256 |  | 0.0 |  | 0.0 |  |
|  |  |  |  |  |  |
